# Towards massively-parallel analytic capabilities for multielectrode recordings

**DOI:** 10.1186/1471-2202-12-S1-P361

**Published:** 2011-07-18

**Authors:** Daniel Gardner, Jason Banfelder, Ajit B Jagdale, Jonathan D Victor

**Affiliations:** 1 Laboratory of Neuroinformatics, Department of Physiology and Biophysics, Weill Cornell Medical College, New York, NY 10021, USA; 2 Institute for Computational Biomedicine, Weill Cornell Medical College, New York, NY 10021, USA; 3 Department of Neurology and Neuroscience, Weill Cornell Medical College, New York, NY 10021, USA

## 

Multielectrode recordings now reliably deliver simultaneous signals from a hundred or more neurons or networks. However, many analytic techniques are presently computationally limited to smaller numbers of signals, severely hampering our ability to relate these neural signals to brain functions including sensation, perception, decision, and action.

To address this imbalance, the Laboratory of Neuroinformatics has begun developing a new open source Neurophysiology Extended Analysis Tool [NEAT]. NEAT leverages existing code bases and new massively-parallel computational technology to enable any multielectrode lab to perform high-throughput informative analyses. The *goal* is to enable neurophysiologists using arrays in cortex and elsewhere to perform analyses online, in real time, that now can be done only offline, and to make possible analyses offline that are now impractical for the number of neurons routinely available to new recording methods. The *method* is to extend our neuroanalysis.org information-theoretic and other spike train analytics to new graphics-processor-derived computational engines [GPUs] supplied on inexpensive drop-in cards.

We have begun by evaluating computational bottlenecks in analytic routines, many information-theoretic, developed for our existing Spike Train Analysis Toolkit [STAToolkit]. The STAToolkit is in wide use, having been distributed via neuroanalysis.org to over 1,700 sites [1]. Central to this effort is appreciation of the specific capabilities and restrictions of GPU-parallel platforms. Our experience, that of others in our project who have used GPUs for other areas of biomedicine, and reports from the EEGLab community, all confirm that generic or library-based solutions show only modest performance gains. We project a greater than order of magnitude speedup that will allow many offline analyses to be performed in real time during experiments, and now-impractical questions to be explored offline in reasonable compute times. For example, pairwise analyses now possible on 10 or fewer neurons may be extendible to 50 or more. We focus on information-theoretic measures and standard pair-wise correlations, JPSTHs, spectra, coherences, and new and significant analyses that present significant loads for multineuron recordings.

To aid communities planning similar GPU-enabled analyses, we note that the complex, structured, and hierarchic GPU architecture requires special optimization strategies:

• decomposing code into >1,000 simultaneous threads is needed to efficiently use the 448 cores on new GPUs,

• data should be loaded into on-chip memory once and re-used, avoiding transfers to other memory layers,

• kernel processes must optimize thread/kernel and thread/block instruction execution in few clock cycles,

• flow control code should control multi-thread warps, not individual threads.
